# Prognostic value of spread through air spaces in operated lung squamous cell carcinoma patients: A meta-analysis

**DOI:** 10.1097/MD.0000000000042940

**Published:** 2025-06-20

**Authors:** Yuan Yuan, Peng Yu

**Affiliations:** a Department of Cardiothoracic Surgery, Xinjiang Military Region General Hospital, Urumqi, People’s Republic of China; b Department of Cardiothoracic Surgery, XPCC Hospital, Urumqi, People’s Republic of China.

**Keywords:** lung squamous cell carcinoma, meta-analysis, prognosis, spread through air spaces, surgery

## Abstract

**Background:**

The presence of spread through air spaces (STAS) predicts poor long-term survival of lung cancer patients. However, the association between STAS and prognosis of operated lung squamous cell carcinoma (LSCC) remains unclear at this time. The aim of this meta-analysis was to further identify the prognostic value of STAS in surgical LSCC patients.

**Methods:**

Several electronic databases were searched up to April 12, 2025 for relevant studies. The primary and secondary outcomes were progression-free survival and overall survival/cancer-specific survival, respectively. The hazard ratios (HRs) and 95% confidence intervals (CIs) were combined and all statistical analyses were conducted by STATA 15.0 software.

**Results:**

A total of 9 studies involving 2884 cases were included and reviewed. The pooled results demonstrated that the presence of STAS was significantly associated with poor progression-free survival (HR = 1.84, 95% CI: 1.57–2.16, *P*< .001). Besides, STAS predicted poorer overall survival (HR = 1.75, 95% CI: 1.23–2.51, *P* = .002) and cancer-specific survival (HR = 1.73, 95% CI: 1.33–2.26, *P* < .001) in surgical LSCC.

**Conclusion:**

Based on current evidence, STAS was identified as a novel and valuable prognostic risk factor for operated LSCC patients. However, more prospective high-quality studies are still needed to verify above findings.

## 1. Introduction

Lung squamous cell carcinoma (LSCC) accounts for around 25% to 30% of non-small cell lung cancer (NSCLC).^[1,2]^ Compared with lung adenocarcinoma, LSCC shows distinct clinicopathologic features such as the higher proportion of advance-stage, male and central cases.^[3–6]^ Besides, LSCC have not benefited from the same therapeutic advances as lung adenocarcinoma, because there is no targeted therapy for LSCC now. Despite of the advances in surgical techniques, chemoradiotherapy and immunotherapy, the overall prognosis remains poor.^[5,6]^ Meanwhile, the reliable and valuable prognostic factors of LSCC are significantly less than those of lung adenocarcinoma. Thus, personalized treatment is still a major challenge for LSCC patients.

Few biomarkers have been reported to be related to therapeutic effects and prognosis of LSCC patients such as the mRNA, miRNA and circulating tumor DNA, and cell-free DNA.^[7–9]^ However, these biomarkers are difficult to obtain and relatively expensive, which limits their clinical application. On the other hand, some easily available parameters based on routine laboratory examinations have been indicated to play a role in predicting postoperative survival of LSCC patients such as the prognostic nutritional index and neutrophil to lymphocyte ratio.^[10–12]^ However, these continuous parameters are unstable and could not directly reflect the disease condition and it is difficult to individually identify the patient’s prognostic risk based on these indicators.

In 2015, spread through air spaces (STAS) was described as a new invasive pattern in the classification of lung cancers proposed by the World Health Organization with the definition as follows: “STAS consists of micropapillary clusters, solid nests, or single cells beyond the edge of the tumor into air spaces in the surrounding lung parenchyma.”^[13]^ STAS is a novel invasion pattern similar to vascular invasion and pleural invasion and was reiterated as a histological feature in the updated classification of thoracic tumor by the World Health Organization in 2021.^[14]^ Up to now, STAS has been verified as a valuable prognostic risk factor in resected lung cancer, especially in lung adenocarcinoma.^[15–18]^ However, the prognostic role of STAS in surgical LSCC patients remains unclear at this time.

Therefore, the aim of this meta-analysis was to further identify the prognostic value of STAS in operated LSCC based on current evidence.

## 2. Materials and methods

This meta-analysis was performed according to the Preferred Reporting Items for Systematic Review and Meta-Analyses 2020.^[19]^

### 2.1. Ethical review

An ethical approval was not applicable because this study is based exclusively on published literature and patient consent was exempt for this type of study.

### 2.2. Literature search

In this meta-analysis, the PubMed, EMBASE and Web of Science electronic databases were searched from inception to April 12, 2025 for relevant studies. The following key words were used during the search: spread through air spaces, STAS, lung, pulmonary, squamous cell carcinoma, survival, prognostic and prognosis. In detail, the specific search strategy was as follows: (spread through air spaces OR STAS) AND (lung OR pulmonary) AND (squamous cell carcinoma) AND (survival OR prognostic OR prognosis). Meanwhile, the MeSH terms and free texts were applied and references cited in included studies were also reviewed for availability.

### 2.3. Inclusion and exclusion criteria

The inclusion criteria were as follows: (1) patients were pathologically diagnosed with primary LSCC; (2) patients received the surgery; (3) the presence or absence of STAS was pathologically detected and the progression-free survival (PFS), overall survival (OS) or (and) cancer-specific survival (CSS) were compared between patients with and without STAS; (4) the hazard ratios (HRs) and 95% confidence intervals (CIs) for the PFS, OS, and CSS were reported or Kaplan–Meier survival curves were provided in articles; (5) high-quality studies with the Newcastle-Ottawa Scale (NOS) score >5.^[20]^

The exclusion criteria were as follows: (1) meeting abstracts, letters, editorials, animal trials or reviews; (2) insufficient, overlapped or duplicated data.

### 2.4. Data extraction

In the current meta-analysis, the following information was collected from each included studies: the name of first author, publication year, country, sample size, number of patients with STAS, tumor-node-metastasis (TNM) stage, endpoint (PFS, OS, CSS), source of HR (reported or estimated), NOS score, and HR with corresponding 95% CI. All the data have been checked and verified by the 2 authors using the Excel tool.

### 2.5. Methodological quality assessment

All included studies were retrospective studies, then the NOS score tool, based on patients selection, comparability, and outcomes, was applied to assess the quality of includes studies and only studies with a NOSC score of 6 or higher.^[20]^

The literature search, selection, data extraction, and quality assessment were all performed by 2 authors (Yuan Yuan and Peng Yu) independently and any disagreement was resolved by team discussion.

### 2.6. Statistical analysis

All statistical analyses were performed using STATA 15.0 software. Heterogeneity among the included studies was evaluated using *I*^2^ statistics and the Q-test. If obvious heterogeneity was observed, representing *I*^2^ > 50% and/or *P* < .1, the random effects model was applied; otherwise, the fixed effects model was used. The HRs and 95% CIs were combined to evaluate the association between STAS and survival of operated LSCC. Considering the similarity between recurrence-free survival (RFS) and PFS, RFS was regarded as PFS during the analysis in this meta-analysis. If the HRs and 95% CIs were not reported in articles, then they would be calculated according to the Kaplan–Meier survival curves through the method described by Tierney et al.^[21]^ Sensitivity analysis was conducted to identify the source of heterogeneity and stability of pooled results. Furthermore, Begg’’s funnel plot and Egger test were conducted to detect publication bias. If significant publication bias was detected, the trim-and-fill method would be applied to evaluate the influence of potentially unpublished papers on the stability of pooled results.^[20]^

## 3. Results

### 3.1. Literature search and selection

**Figure 1.**
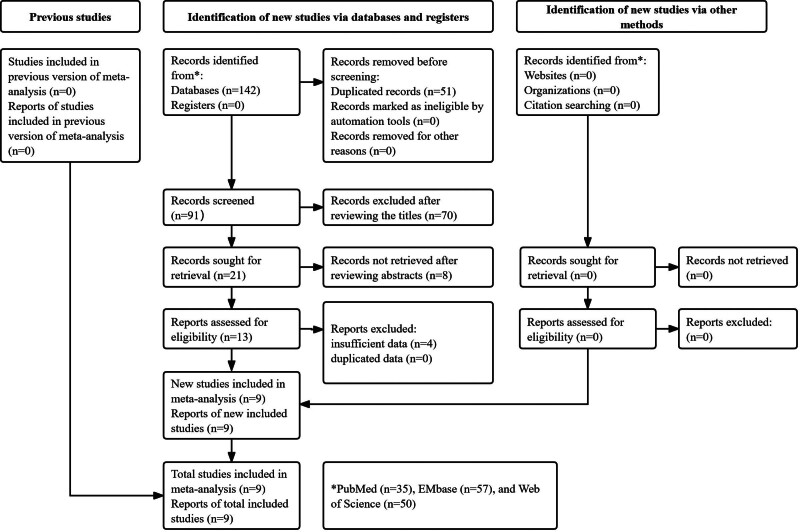
Prisma flow diagram of this meta-analysis.

Initially, 142 records were identified from the 3 databases and 51 duplicated records were removed. After screening the titles and abstracts, 78 publications were excluded and the full texts of remaining 9 studies were reviewed carefully. Eventually, 9 studies were included in this meta-analysis^[22–30]^ and the detailed process was presented in Fig. 1.

### 3.2. Basic characteristics of included studies

Among the 9 studies, a total of 2884 patients were enrolled with the sample size ranged from 79 to 681. In the studies by Stogbauer et al and Yu et al,^[26,27]^ 2 independent study populations were separately reviewed, thus we regarded them as “two studies” during the analysis process in our meta-analysis, respectively. All included studies were high-quality studies with a NOS score ≥ 6. Other detailed information was shown in Table 1.

**Table 1:** Basic characteristics of included studies.

Author	Year	Country	Sample size	Number of patients with STAS	TNM stage	Endpoint	Source of HR	NOS
Kadota^[22]^	2017	Japan	216	87	I–IV	PFS	R	7
Lu^[23]^	2017	Japan	445	132	I–III	PFS, CSS, OS	R	7
Yanagawa^[24]^	2018	Japan	220	42	I, II–III	PFS, OS	R	7
Dagher^[25]^	2022	France	241	86	I–III	OS	R	7
Stogbauer^[26]^	2022	Germany	335	–	I–IV	PFS, OS	R	6
Stogbauer^[26]^	2022	Germany	346	–	I–IV	CSS, OS	R	6
Yu^[27]^	2022	China	324	114	I–III	PFS	R	7
Yu^[27]^	2022	China	216	82	I–III	PFS	E	7
Zombori-Tóth^[28]^	2023	Hungary	220	57	I–III	PFS	R	7
Akcam^[29]^	2024	Turkey	79	26	I–IIA	PFS	R	6
Senchukova^[30]^	2024	Russia	242	–	I–III	PFS	R	6

CSS = cancer-specific survival, E = estimated, HR = hazard ratio, NOS = Newcastle-Ottawa Scale, OS = overall survival, PFS = progression-free survival, R = reported, STAS = spread through airspaces, TNM = tumor-node-metastasis.

### 3.3. The association between STAS and PFS of surgical LSCC patients

**Figure 2.**
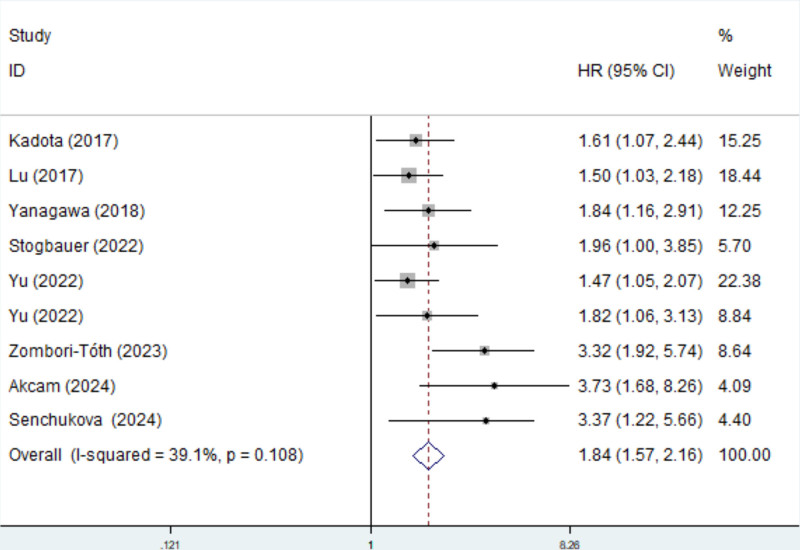
The association between STAS and PFS in operated LSCC. LSCC = lung squamous cell carcinoma, PFS = progression-free survival, STAS = spread through air space.

Eight studies explored the predictive role of presence of STAS for PFS in operated LSCC.^[22–24,26–30]^ The pooled results demonstrated that the presence of STAS predicted significantly poorer PFS (HR = 1.84, 95% CI: 1.57–2.16, *P* < .001; *I*^2^ = 39.1%, *P* = .108) (Fig. 2).

### 3.4. The association between STAS and OS and CSS of surgical LSCC patients

**Figure 3.**
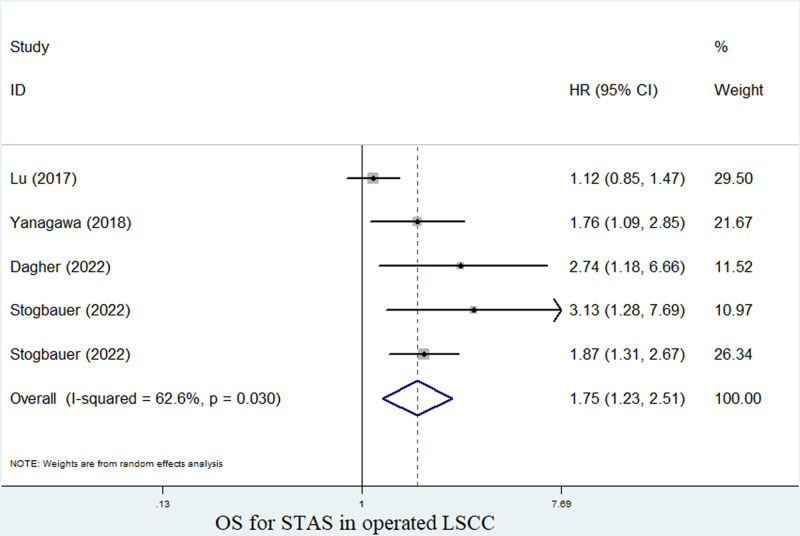
The association between STAS and OS in operated LSCC. LSCC = lung squamous cell carcinoma, OS = overall survival, STAS = spread through air space.

**Figure 4.**
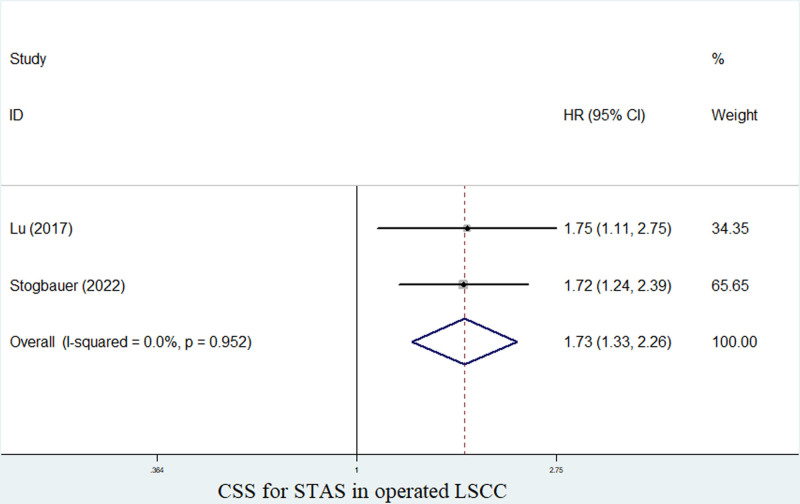
The association between STAS and CSS in operated LSCC. CSS = cancer-specific survival, LSCC = lung squamous cell carcinoma, STAS = spread through air space.

Besides, 4 and 2 studies explored the relationship between STAS and OS^[23–26]^ and CSS^[23,26]^ of surgical LSCC patients, respectively. The pooled results manifested that the presence of STAS was obviously related to poor OS (HR = 1.75, 95% CI: 1.23–2.51, *P* = .002; *I*^2^ = 62.6%, *P* = .030) (Fig. 3) and CSS (HR = 1.73, 95% CI: 1.33–2.26, *P* < .001; *I*^2^ = 0.0%, *P* = .952) (Fig. 4).

### 3.5. Sensitivity analysis and publication bias

**Figure 5.**
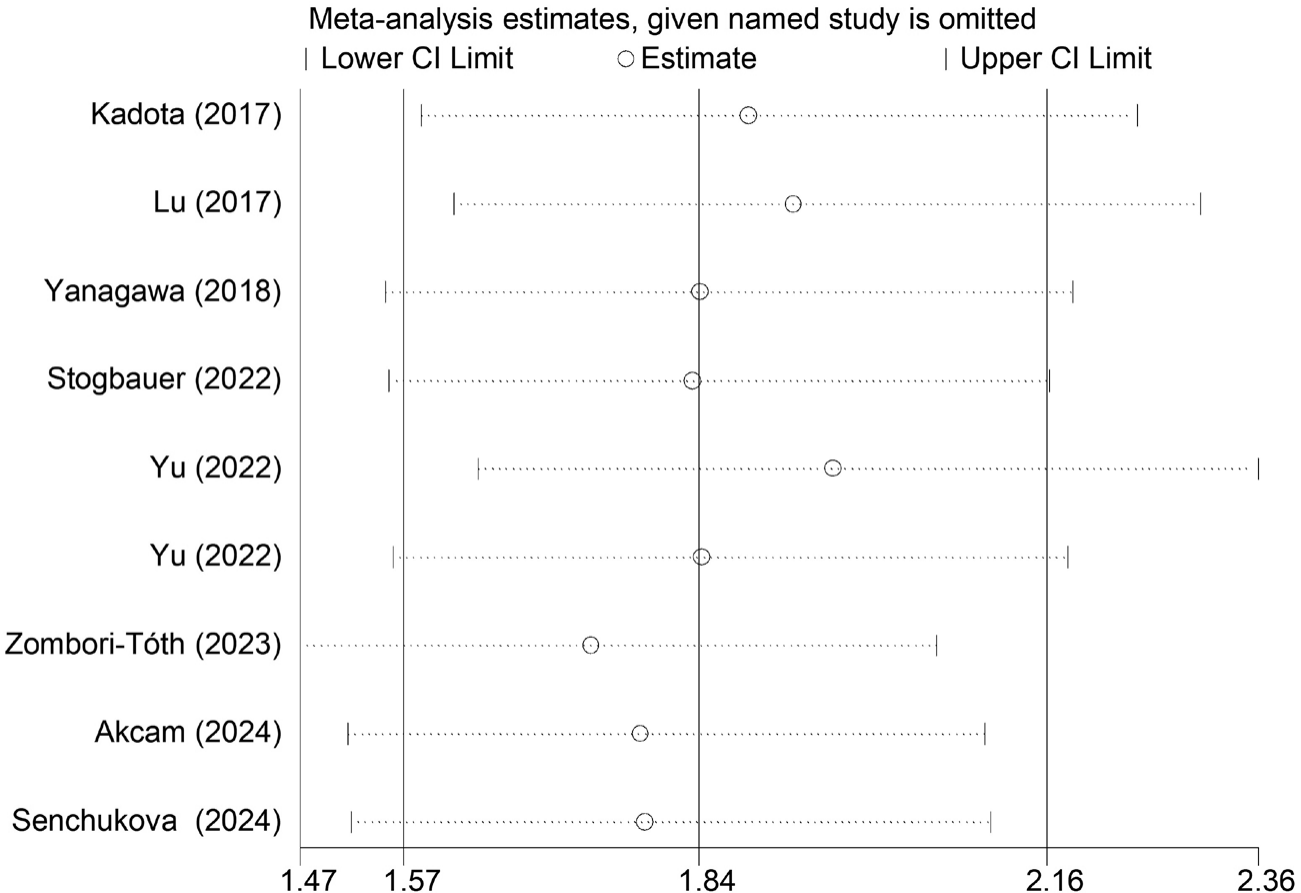
Sensitivity analysis for the association between STAS and PFS in operated LSCC. LSCC = lung squamous cell carcinoma, PFS = progression-free survival, STAS = spread through air space.

**Figure 6.**
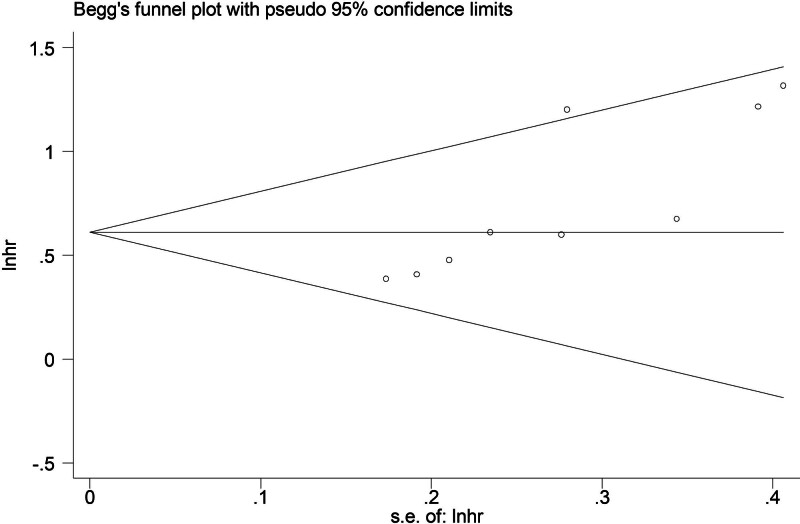
Begg funnel plot for the association between STAS and PFS in operated LSCC. hr = hazard ratio, LSCC = lung squamous cell carcinoma, PFS = progression-free survival, se = standard error, STAS = spread through air space.

**Figure 7.**
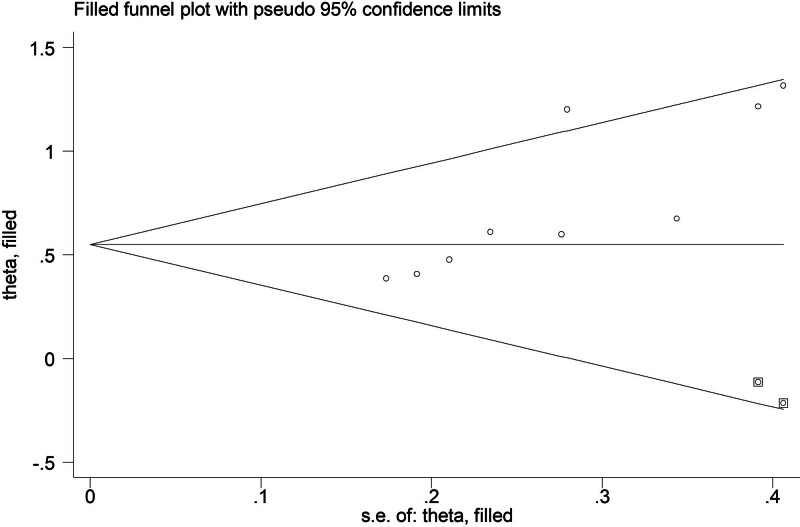
Filled funnel plot for the association between STAS and PFS in operated LSCC. LSCC = lung squamous cell carcinoma, PFS = progression-free survival, se = standard error, STAS = spread through air space.

According to the sensitivity analysis for the PFS (Fig. 5), the pooled results of this meta-analysis were stable and reliable. However, the Begg funnel plot (Fig. 6) and Egger test (*P* = .004) indicated that obvious publication bias existed. Therefore, the trim-and-fill method was applied and one potentially “unpublished” study was detected (Fig. 7), but this study did not cause an significant impact on the overall results (filled HR = 1.73, 95% CI: 1.49–2.02, *P* < .001 for fixed effects model; filled HR = 1.79, 95% CI: 1.42–2.25, *P* < .001 for random effects model).

## 4. Discussion

The current meta-analysis demonstrated the STAS was significantly associated with prognosis in surgical LSCC and patients with STAS experienced obviously poorer postoperative survival than patients without STAS did. Therefore, STAS could serve as a novel and valuable prognostic factor in operated LSCC patients and contribute to the formulation of therapy strategy. However, due to the limitations existed in included studies, more prospective and high-quality studies with larger sample sizes are still needed to further verify above findings.

The clinical role of STAS in lung cancer has been reported by several meta-analyses up to now. Pyo et al included 47 studies and manifested that the incidence rate of STAS in LSCC was 33.8% (27.3–41.1%).^[31]^ Besides, STAS was obviously associated with a higher incidence rate of venous, visceral pleural and lymphatic invasion, anaplastic lymphoma kinase mutation and ROS1 rearrangement, and the presence of STAS predicted worse RFS (HR = 2.372, 95% CI: 2.018–2.788) and OS (HR = 2.119, 95% CI: 1.811–2.480) in NSCLC.^[31]^ Yang et al included 11 relevant studies involving 5097 stage I lung adenocarcinoma patients and demonstrated that the presence of STAS was significantly associated with poorer RFS (HR = 1.95, 95% CI: 1.58–2.31, *P* < .01) and OS (HR = 2.04, 95% CI: 1.60–2.48, *P* < .01).^[32]^ Meanwhile, they also indicated that sublobectomy should be carefully selected for stage I lung adenocarcinoma patients with positive STAS because of the significantly high risk of recurrence (HR = 6.92, 95% CI: 1.64–12.18, *P* < .01).^[32]^ This study was the first meta-analysis to identify the prognostic value of STAS in LSCC and indicated the predictive role of the presence of STAS for worse prognosis of LSCC patients, although we could not perform more detailed analyses due to the insufficient data.

In the study by Yanagawa et al., although the positive association between the presence of STAS and poor survival was observed in the entire cohort, different results were identified between stage I and II/III patients.^[24]^ In detail, the presence of STAS predicted poor PFS (HR = 3.27, 95% CI: 1.70–6.29, *P* < .001) and OS (HR = 3.01, 95% CI: 1.54–5.89, *P* = .001) only in stage I patients, and nonsignificant association of STAS with PFS (HR = 1.11, 95% CI: 0.56–2.21, *P* = .756) or OS (HR = 1.11,95% CI: 0.54–2.27, *P* = .768) of stage II/III LSCC patients was detected.^[24]^ Thus, the tumor stage may affect the prognostic value of STAS in operated LSCC, which should be further explored in future relevant studies. Besides, some other important parameters may also affect the predictive role of STAS for long-term survival of LSCC such as the sex, histological subtype and differentiation grade. Furthermore, some early-stage LSCC patients may receive the limited resection, but STAS has been reported to occur in about 20% of stage I LSCC patients.^[24]^ Whether lobectomy is more appropriate for LSCC patients with the presence of STAS remains unclear. It is also needed to further determine the necessity of postoperative adjuvant therapy in stage IA LSCC patients.

There are several limitations in our meta-analysis. First, all included studies are retrospective and the overall sample size is relatively small, which might cause some bias. Secondly, we tried several times to contact authors of included studies for original data. However, we did not get a positive response from the authors. Thus, we were unable to conduct more specific analyses and explore the impact of some important parameters such as the TNM stage and postoperative adjuvant therapy on the prognostic role of STAS in surgical LSCC patients. Thirdly, significant heterogeneity for the OS was observed (*I*^2^ = 62.6%, *P* = .030). However, we were unable to further identify the source of heterogeneity and potential confounding factors. Therefore, more prospective and big-sample studies with specific subgroup analysis are needed to further validate the prognostic value and clinical application of STAS in surgical LSCC patients.

## 5. Conclusion

STAS could serve as a valuable and reliable prognostic risk factor in surgical LSCC and patients with STAS are manifested to experience significantly worse prognosis. However, more high-quality studies with larger sample sizes are still needed to verify above findings.

## Author contributions

**Conceptualization:** Yuan Yuan, Peng Yu.

**Data curation:** Yuan Yuan.

**Formal analysis:** Yuan Yuan.

**Investigation:** Yuan Yuan.

**Methodology:** Peng Yu.

**Software:** Yuan Yuan, Peng Yu.

**Supervision:** Peng Yu.

**Visualization:** Yuan Yuan.

**Writing – original draft:** Yuan Yuan.

**Writing – review & editing:** Peng Yu.
